# Detecting Hypoxia Through the Non-Invasive and Simultaneous Monitoring of Sweat Lactate and Tissue Oxygenation

**DOI:** 10.3390/bios14120584

**Published:** 2024-11-30

**Authors:** Cindy Cheng, Sayan Ganguly, Pei Li, Xiaowu Tang

**Affiliations:** Department of Chemistry, University of Waterloo, 200 University Ave West, Waterloo, ON N2L 3G1, Canada; c29cheng@uwaterloo.ca (C.C.); sganguly@uwaterloo.ca (S.G.); p2li@uwaterloo.ca (P.L.)

**Keywords:** hypoxia, wearable sensor, sweat lactate, tissue oxygenation

## Abstract

Hypoxia, characterized by inadequate tissue oxygenation, may result in tissue damage and organ failure if not addressed. Current detection approaches frequently prove insufficient, depending on symptoms and rudimentary metrics such as tissue oxygenation, which fail to comprehensively identify the onset of hypoxia. The European Pressure Ulcer Advisory Panel (EPUAP) has recognized sweat lactate as a possible marker for the early identification of decubitus ulcers, nevertheless, neither sweat lactate nor oxygenation independently provides an appropriate diagnosis of hypoxia. We have fabricated a wearable device that non-invasively and concurrently monitors sweat lactate and tissue oxygenation to fill this gap. The apparatus comprises three essential components: (i) a hydrogel-based colorimetric lactate biosensor, (ii) a near-infrared (NIR) sensor for assessing tissue oxygenation, and (iii) an integrated form factor for enhanced wearability. The lactate sensor alters its hue upon interaction with lactate in sweat, whereas the NIR sensor monitors tissue oxygenation levels in real-time. The device underwent testing on phantom exhibiting tissue-mimicking characteristics and on human sweat post aerobic and anaerobic activities. Moreover, the device was demonstrated to be capable of real-time “on-body” simultaneous monitoring of sweat lactate spikes and tissue oxygenation (StO_2_) drops, which showed strong correlation during a hypoxia protocol. This innovative technology has a wide range of potential applications, such as post-operative care, sepsis detection, and athletic performance monitoring, and may provide economical healthcare solutions in resource-limited regions.

## 1. Introduction

Wearable sensors have become a significant growing sector for consumer, sports, and military applications, providing distinctive opportunities for in situ, real-time, and non-invasive health and performance monitoring [1]. The consumer electronics industry is presently saturated with instances of wearable technology in wrist-worn, textile, or strap-mounted configurations, with inflexible active materials and cumbersome power sources [2,3]. Available measures are typically restricted to elevated physiological signals, including temperature, heart rate, skin conductance, and respiratory rate [4]. Recent advancements in conformal and flexible electronics have yielded remarkable demonstrations of research-grade prototypes possessing comparable physiological measurement capabilities, yet with significantly different form factors [5]. Simultaneously, considerable focus is directed towards monitoring analyte presence in biofluids, hence broadening the scope of information obtainable from the wearer [6,7,8]. Sweat is an optimal subject for analysis owing to its suitability for non-invasive collection [9]. Numerous sweat-sensing devices have been showcased in the forms of patches, watches, and temporary tattoos, capable of measuring analytes including lactate, glucose, ammonia, ethanol, trace metals, hydration biomarkers, pH, and other metal ions found in sweat [10]. Complex systems that integrate measurements of many biomarkers and physiological signals on a single platform have also been documented, demonstrating the possibility for completely autonomous integrated systems [11,12,13,14].

Sweat presents a viable substitute for blood in routine analyses conducted on the sports field. Sweat is produced during physical exercise, its collection is non-invasive, and it contains numerous biomarkers indicative of physiological states, such as lactate [15]. Although recent research suggests that sweat lactate may effectively substitute blood analysis for assessing sports performance, this assertion remains contentious [16]. Numerous lactate sensors have been documented in the literature, aiming to deliver reliable on-body data for physiological evidence [17], including colorimetric, electrochemiluminescent, and electrochemical measurements [18]. Promphet et al. have created a colorimetric textile-based sensor for lactate monitoring on the body [19]. Chen et al. have introduced a versatile electrochemiluminescence platform capable of responding to concentrations up to 20 mM [20]. The sensor was effectively utilized in two on-body tests, yielding several discrete results. Nonetheless, the deterioration and/or leaching of the luminophore material over prolonged measurements are a concern, since it may result in erroneous outcomes over time.

Lactate is a by-product of glycolysis, produced during anaerobic glucose metabolism in response to the body’s elevated energy requirements. It is a crucial indicator for assessing oxidative stress levels, muscular health, and tissue hypoxia [21]. The net lactate concentration in these biofluids is significantly influenced by the physiological condition and the location of anaerobic metabolism within the human body [22]. Lactate monitoring is crucial for some populations, particularly those susceptible to hypoxic situations, such as athletes and military personnel, to enhance training and endurance performance [23]. Elevated lactate levels in the body can rapidly disrupt fluid pH and lead to several adverse health consequences, including seizures, sepsis, renal failure, malignancies, cerebral stroke, and panic disorders [24]. The lactate concentration in eccrine sweat is influenced by various factors, including the kind of effort, duration, intensity, and sweat rate [25]. Medium-intensity activity for a brief period, or heat-induced perspiration, might produce a lactate concentration of up to 25 mM, whereas strenuous outdoor activities may provide concentrations as high as 115 mM [26,27,28]. Conversely, it has been demonstrated that sweat lactate concentration inversely correlates with sweat rate [29]. Evidence indicates that sweat lactate levels exhibit minor variation with age [30], sex [31], and sampling site (attributable to differences in sweat gland density) [32]. In addition to eccrine sweat glands, two further types of sweat glands have been identified in the body: apocrine and apoeccrine [33]. Although these glands produce perspiration, lactate is rarely found in it, thus we focus on the forearm eccrine glands, a common lactate testing site. Alam et al. have determined that blood lactate concentrations in healthy individuals at rest range from 0.5 to 2 mM [34]. During moderate to medium-intensity exercise, blood lactate concentration exhibits no substantial variation in magnitude and does not correlate well with sweat lactate concentration [35]. Goodwin et al. demonstrated that upon surpassing the lactate threshold (LT), blood lactate concentration increases exponentially, potentially reaching a peak of 11 mM [33]. Karpova et al. conducted a study demonstrating a favorable association between lactate levels in blood and perspiration after high-intensity exercise [36]. Laboratory and commercial sensing platforms use lactate oxidase (LOx) or dehydrogenase. Previous colorimetric skin-interfacing wearable sensors convey sweat to a lactate-specific enzyme assay area via complex serpentine microfluidic channels [37,38]. The lactate present in the sweat undergoes a reaction that produces a colored compound, the intensity of which is measured by mobile image analysis [39,40].

This study delivers an innovative method for the non-invasive detection of hypoxia by concurrently monitoring sweat lactate and tissue oxygenation, a combination that, to our knowledge, has not been previously reported. Although prior research has examined tissue oxygenation and lactate levels separately as indicators of hypoxia, their simultaneous real-time assessment provides a more thorough comprehension of the condition. Our engineered wearable device seamlessly combines a colorimetric lactate biosensor with a near-infrared (NIR) sensor, delivering real-time, non-invasive analysis of metabolic and tissue oxygenation conditions. The innovation resides in the integration of these two indicators into a singular device, facilitating improved precision in identifying hypoxic states and providing expanded therapeutic applications, including post-operative management and athletic surveillance. This dual-sensing technique enhances hypoxia diagnosis and facilitates economical, accessible healthcare solutions, especially in resource-constrained environments.

## 2. Materials and Methods

Materials. Sodium L-lactate (~98%), Alginate (sodium salt), Calcium chloride (CaCl_2_), L-Lactate Oxidase (LOX), Horseradish peroxidase (HRP), and 3,3′,5,5′-Tetramethylbenzidine (TMB) were purchased from Sigma-Aldrich, Canada. Cellulose nanocrystal (CNC) was purchased from CelluForce Inc., Montreal, QC, Canada. Sodium hydroxide (NaOH, ≥97.0%) was purchased from Fisher Scientific (Canada). All aqueous solutions were prepared using highly purified water from a Millipore Milli-Q water system (≥18 MΩ cm). All chemicals were used without further purification.

UV-vis spectroscopy. The absorbance of lactate-sensing hydrogels was measured at 570 nm using a UV–vis spectrophotometer (Molecular Devices SpectraMax M2, Marshall Scientific, Hampton, NH, USA).

Preparation of lactate-sensing hydrogel. A homogenous alginate solution at 4 wt% was prepared by adding alginate powder to PBS (pH 7.4) and stirring for 2 h at 60 °C. The solution was then cooled to room temperature before use. CNC powder was added to DI water and sonicated briefly to make a homogeneous 2 wt% suspension. TMB was added drop-wise to the CNC suspension until the surface of the CNC nanoparticles was saturated. The TMB-loaded CNCs were collected by centrifugation and the precipitate was resuspended in DI water. The alginate and TMB/CNC solutions were then mixed at a 1:1 ratio. Right before performing the colorimetric assay, LOX and HRP solutions were added. The final concentrations in the pre-gel solution were 2 wt% alginate, 1 wt% TMB/CNC, 50 μg/mL LOX, and 2 μg/mL HRP. The pre-gel solution was then pipetted into a mold, covered with an ion-permeable membrane, and submerged in a 3 wt% CaCl_2_ solution to crosslink.

NIR sensor electronics and readout software. The optical readout electronics comprising a small PCB board (3 cm × 2 cm) with the driving circuits of two photodetectors (PDs), two light-emitting diodes (LEDs), and a USB interface, along with its driver and application software, were donated by Mespere Lifesciences, Inc. (Waterloo, ON, Canada). The LEDs and PDs operate at wavelengths of 660 nm and 905 nm. The optical electronics are part of medical devices that have received regulatory approval for human use in Canada and the United States of America.

Enclosure for device integration. An enclosure was designed using SolidWorks and then 3D printed via Direct Light Processing (DLP) using Acrylonitrile Butadiene Styrene (ABS) resin (Photon Mono 4K, Anycubic, Shenzhen, China). The enclosure consists of two parts: one part houses the optical readout electronics, which is isolated from the second part containing hydrogel chambers, using a transparent waterproof double-sided adhesive.

Optical tissue phantom. Pro-Jet™ 900 NP dye (4 mg) was weighed out in a scintillation vial, then 20 mL ethanol was added, and the mixture was sonicated for 30 min to make a 0.2 mg/mL dye solution. TiO_2_ powder (2 g) was then added to the Pro-Jet™ 900 NP solution (20 mL) and sonicated for 30 min. A high-speed mixer at 1200 RPM was used to mix Castin’ Craft Clear Polyester Resin (2 L) with the TiO_2_/Pro-Jet™ 900 NP solution (20 mL), and followed by the addition of Castin’ Craft^®^ Clear Catalyst (50 drops/L). The mixture was then degassed in a vacuum oven to remove air bubbles and carefully poured into a polyvinyl chloride (PVC) mold with a diameter of 15 cm and height of 10 cm. Further degassing was done to remove any remaining bubbles. The mixture was left 48 h to solidify before the solid-state tissue phantom was released from the mold and polished. The optical phantom possesses optical absorption and scattering properties similar to those of human tissue.

## 3. Results and Discussions

### 3.1. Lactate-Sensing Hydrogel

**Figure 1:**
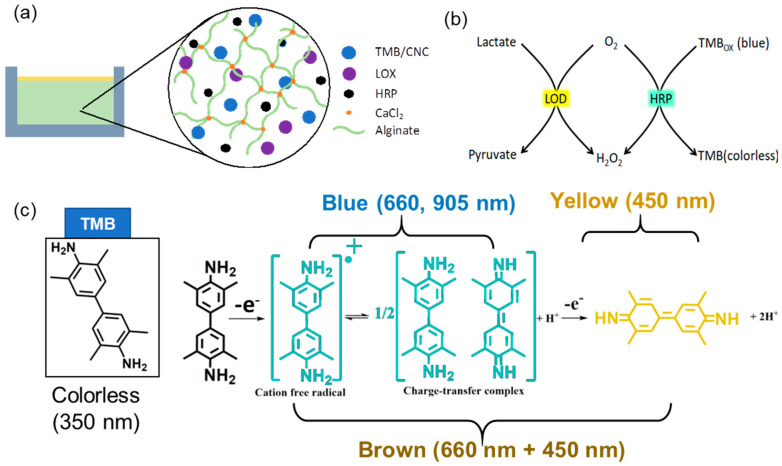
Schematic illustration of (**a**) the lactate-sensing hydrogel composition and (**b**) the colorimetric sensing mechanism. Oxidation of lactate, catalyzed by LOX, generates H_2_O_2_ as a byproduct; TMB reacts with H_2_O_2_, catalyzed by HRP, results in a color change. (**c**) Optical absorption peaks of TMB (350 nm) and TMB_ox_ (one-electron oxidation, 660 and 960 nm; two-electron oxidation, 450 nm).

Figure 1a illustrates the construction of the alginate-based hydrogel and its lactate detection mechanism. The hydrogel was created by integrating essential components: TMB-loaded cellulose nanocrystals (CNC), lactate oxidase (LOX), horseradish peroxidase (HRP), and sodium alginate. Alginate was selected for its biocompatibility and capacity to create a durable hydrogel matrix through ionic crosslinking with calcium ions (Ca^2^), so ensuring structural integrity and preserving the active components inside the gel. LOX and HRP, encapsulated inside the matrix, are crucial for the enzymatic cascade required for lactate detection. To prevent the leaching of TMB, which is a small molecule, TMB was first loaded onto negatively charged CNC. CNCs are nanoparticles about 20 nm in diameter and 200 nm in length [41]. LOX and HRP are macromolecules with molecular weights of approximately 80 kDa and 40 kDa, respectively, which considerably exceed the sizes of small analytes like lactate (90 Da) and oxygen (32 Da). The alginate-based hydrogel used in this investigation was engineered to entrap the enzymes while allowing the efficient transport of small analyte molecules. The mesh size of the alginate network was adjusted by optimizing the alginate content and crosslinking density during preparation [42,43]. Furthermore, the enzymes LOX and HRP were pre-mixed with alginate prior to gelation. Non-covalent interactions, such as hydrogen bonding and electrostatic interactions between the enzymes and alginate, are expected to be strong. Consequently, the enzymes are further retained through polymer entanglement. No noticeable leakage of LOX and HRP was observed.

Upon lactate diffusion into the hydrogel, LOX facilitates its oxidation to pyruvate and hydrogen peroxide (H_2_O_2_) (Figure 1b). The presence of H_2_O_2_ is essential, serving as a substrate for HRP, which oxidizes TMB from its colorless reduced form to its blue oxidized form (TMB_ox_), with the intensity of the blue color directly correlating to lactate concentration, facilitating visual or spectrophotometric detection. The colorimetric transition depicted in Figure 1c entails TMB initially absorbing at 350 nm in its reduced state, subsequently undergoing oxidation to generate a blue cation/free radical, which exhibits absorption peaks at 660 nm and 905 nm. This intermediate can undergo further oxidation to form a brown charge-transfer complex, which absorbs at both 660 and 450 nm, and ultimately transitions into a fully oxidized yellow state, absorbing at 450 nm [44]. The specific color transitions, from colorless to blue, brown, and yellow, directly indicate lactate concentration, enabling real-time, non-invasive monitoring of lactate levels in sweat.

### 3.2. UV-Vis Spectroscopic Validation of the TMB Assay in Hydrogel

Unlike the commonly performed liquid-based TMB assay, the rate of reaction in a hydrogel, which is less studied, is dictated by mass transfer. The enzymes, LOX and HRP, and the TMB/CNCs are trapped in the solid phase of the gel. The diffusion rates of lactate and H_2_O_2_ in the hydrogel are controlled by alginate concentration and the crosslinking density. It is essential to fabricate hydrogels with enzymes (LOX and HRP) and chromogenic dyes (TMB) uniformly distributed in the gel matrix to prevent TMB from leaching out of while maintaining its reactivity, and to ensure a capacity and rate of colorimetric change suitable for monitoring sweat lactate. Before integration into the wearable device, lactate-sensing hydrogels were first formed in a 96-well plate to evaluate their performance using a UV-vis spectrometer.

**Figure 2:**
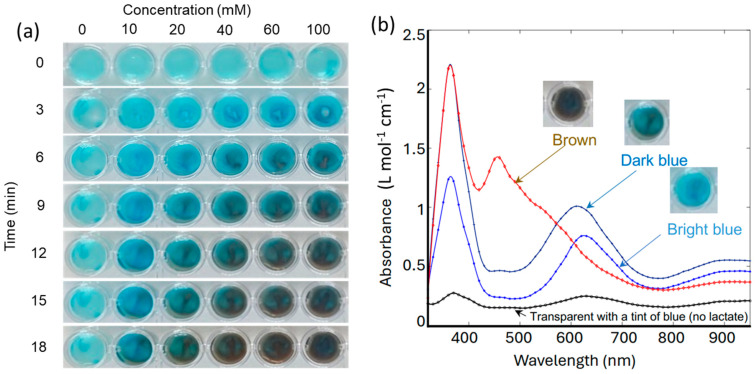
(**a**) Images of gels in a multi-well plate in response to various lactate concentrations at various time points. (**b**) Representative optical absorption spectra of gels in response to lactate.

As shown in Figure 2a, a row of wells was filled with the lactate-sensing hydrogels, dosed with various concentrations of lactate, and photographed every 3 min. For dosing, a drop (50 uL) of lactate solution in PBS was added to each well. The lactate concentrations were 0, 10, 20, 40, 60, 100 mM, respectively. Higher concentrations of lactate were observed to induce more significant and faster colour change. Figure 2b shows the representative absorption spectra and their corresponding colours. In the absence of lactate, the gel appeared transparent with a tint of blue, due to minimal absorption at 660 nm and 905 nm. The data presented in Figure 2b indicate that the rate of change in light absorption at 660 nm is directly proportional to the increase in lactate concentration, as this wavelength corresponds to the blue cation/free radical form of TMB, which is formed during the initial oxidation stage [45]. As lactate concentration increases further, the absorption at 905 nm increases as the hydrogel transitions to a brown charge-transfer complex [46]. This dual-wavelength analysis allows for the precise quantification of lactate concentration based on the spectral changes observed during the oxidation of TMB. The fully oxidized TMB species ultimately absorbs at 450 nm, indicating complete lactate saturation in the system, which should be avoided during real-time sensing. The hydrogel formulation was deemed optimal since the rate of colorimetric change is adequate, with a clear color transition from transparent (no lactate) to bright or dark blue in about 10 min at lactate concentrations up to 60 mM. For reference, sweat lactate levels range from 5 to 20 mM during resting or low activity and can reach up to 50 mM during intense physical activity. This detailed spectrometric analysis confirms that the hydrogel can reliably detect varying lactate concentrations by measuring the rate of change in light absorption at 660 nm and 905 nm, enabling the real-time, non-invasive monitoring of lactate levels.

### 3.3. Device Design and Fabrication

**Figure 3:**
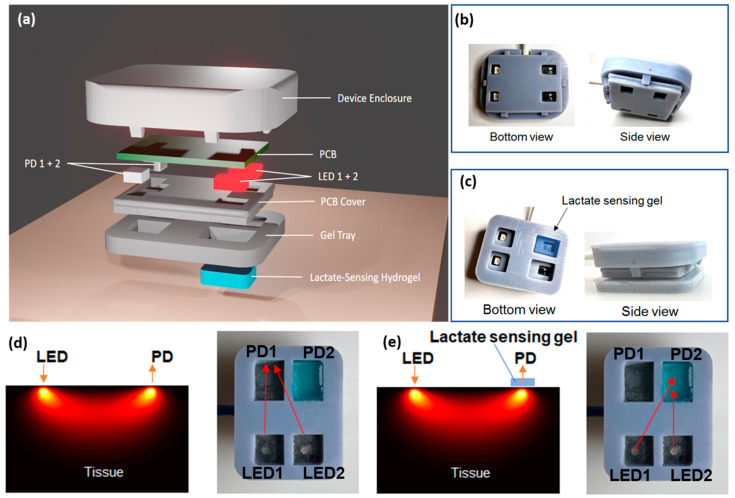
(**a**) An exploded view of the device prototype. Pictures of an assembled wearable device, (**b**) without, and (**c**) with the gel. Optical pathways from LEDs to PDs (**d**) without and (**e**) with the lactate sensing hydrogel on tissue. Left: simulation in tissue; right: bottom view of the wearable device.

The device depicted in Figure 3a incorporates modern sensor technologies into a compact, wearable format for real-time, non-invasive, and concurrent monitoring of lactate and tissue oxygenation. It consists of two major functional components: the hydrogel-based lactate sensor and the near-infrared (NIR) sensor, both crucial for monitoring biochemical and physiological alterations in the body [47]. The device enclosure was precisely designed to facilitate secure integration of various components, guaranteeing durability, adaptability, and user-friendliness for ongoing wearable monitoring. This enclosure was 3D printed using a DLP printer, ensuring easy customization and high-resolution fabrication. The device enclosure comprised three essential parts: a top cover, a PCB cover, and a gel tray, all of which were assembled to provide structural integrity and facilitate the placement of the hydrogel sensor.

The NIR sensor comprises two light emitter (LED1 and LED2) and photodetector (PD1 and PD2) pairs, specifically engineered to engage with the hydrogel sensor and tissue for precise data gathering. The top cover and the PCB cover click together to form a waterproof shell around the electronics (Figure 3b). Four openings on the PCB cover allowed light to pass through and were sealed with a transparent waterproof adhesive, which also enabled the secure attachment of the gel tray (Figure 3c). The gel tray with four rectangular chambers was designed specifically to accommodate the lactate-sensing hydrogel, allowing for easy sample change and optimal interaction with sweat. The snug fit of the enclosure with the PCB board, and the rims separating the gel chambers, are critical to prevent light leakage and environmental light interference.

### 3.4. Principle of Operation

The optical pathways between the LED/PD pairs are illustrated in Figure 3d,e. When the device is placed on skin, PD1 measures the attenuation of light emitted by LED1 through skin and body tissue underneath, while PD2 measures the attenuation of light emitted by LED2 through both tissue and the hydrogel. LED1 and LED2 were turned on alternately. The following equations describe the light intensities detected by the PD1 (*I*_1_) and PD2 (*I*_2_):

(1) $$I_{1}=I_{0}\emptyset \left(\mu_{a},\mu_{s}\right)$$

(2) $$I_{2}=I_{0}\emptyset \left(\mu_{a},\mu_{s}\right)\cdot e^{-\mu_{a,g}l_{g}}$$

where *I*_0_ is the light intensity emitted by the LED, $\mu_{a}$, $\mu_{s}$, $\emptyset \left(\mu_{a},\mu_{s}\right)$ are the absorption coefficient, scattering coefficient, and overall light attenuation of the tissue underneath the device, and $\mu_{a,g}$ and $l_{g}$ are the absorption coefficient and thickness of the lactate-sensing hydrogel. The light intensity emitted by LED1 and LED2 is set to be identical, *I*_0_, by the LED driver electronics.

By using the pair of LED1 and PD1 alone (Figure 3d, Equation (1)), without the lactate-sensing hydrogel in the optical pathway, the NIR sensor can accurately detect the tissue oxygenation level (StO_2_) and blood volume index (BVI) [48], without being affected by lactate. In human blood, oxygen is primarily carried by the protein hemoglobin. Blood oxygenation (SpO_2_) is, thus, defined as the ratio of oxygenated hemoglobin (HbO_2_) to total hemoglobin (HbT), while tissue oxygenation (StO_2_) is the average of arterial and venous blood SpO_2_. HbO_2_ and Hb have distinctively different absorption spectra, dominating over other chromophores found in tissue in the near-infrared (NIR) window. This allows for the use of NIR spectroscopy (NIRS) to determine their concentrations [49]. In our device, each LED and PD can emit and detect at both 660 nm and 905 nm. By measuring *I*_1_ at the two wavelengths, the concentrations of both Hb and HbO_2_ can be determined. Subsequently, HbT (the sum of Hb and HbO_2_) and StO_2_ can be calculated. HbT is proportional to the blood volume index (BVI).

Due to the close proximity of the two LED/PD pairs, the light attenuation by tissue, $\emptyset \left(\mu_{a},\mu_{s}\right)$, between LED1/PD1 and LED2/PD2, can be assumed to be the same. Therefore, by taking the ratio of *I*_2_ to *I*_1_ (i.e., Equation (2) divided by Equation (1)), the absorption coefficient of the lactate-sensing hydrogel, $\mu_{a,g}$, can be calculated as shown in the equation below:

(3) $$\mu_{a,g}=-\ln\left(I_{2}/I_{1}\right)/l_{g},$$

where the hydrogel thickness ($l_{g})$ is molded to be 2 mm. Since $\mu_{a,g}$ is linearly proportional to the amount of TMB_ox_ generated in the sensing gel in response to lactate, we were able to quantitatively measure lactate concentration, without interference from the light attenuation by the skin and tissue. The capability of our device to monitor both a biochemical indicator (lactate) in sweat and physiological parameters (StO_2_ and BVI) in tissue concurrently, without cross-talk, provides a comprehensive understanding of hypoxia and metabolic stress in real time.

### 3.5. Device Validation on a Solid-State Tissue Phantom

Once assembled, the integrated device was first tested on a phantom with tissue-mimicking optical properties to characterize its performance. This phantom mimicked the optical characteristics of human skin and tissue, ensuring that the device’s measurements of light attenuation, tissue oxygenation, and lactate detection were reliable before proceeding to human studies. This initial characterization phase was critical in validating the device’s ability to simultaneously monitor sweat lactate and tissue oxygenation under controlled conditions.

**Figure 4:**
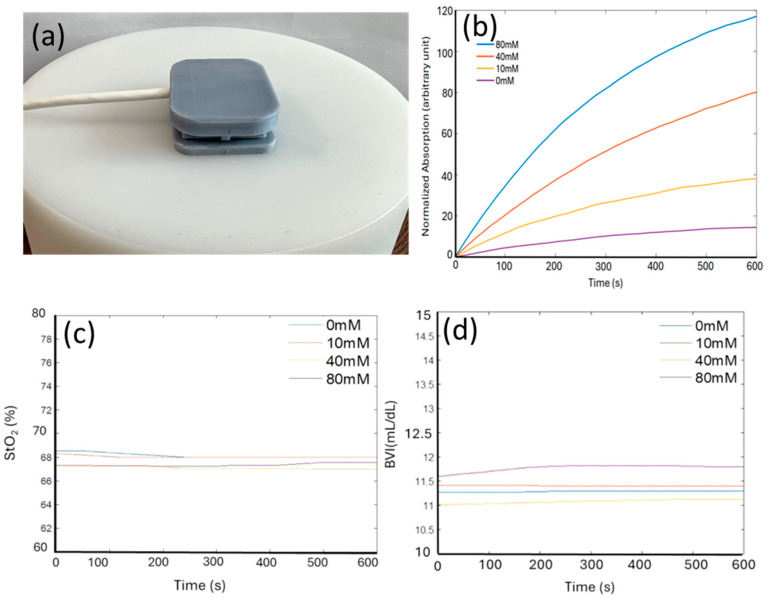
(**a**) Picture of the device placed on top of an optical phantom with optical properties mimicking those of human tissue (StO_2_ = 68%). (**b**) Normalized absorption of the lactate sensing hydrogels, extracted from the optical readout, in response to various lactate solutions (0, 10, 40, 80 mM) in phosphate buffer (pH 6.0) respectively. (**c**,**d**) Stable StO_2_ and BVI reading regardless of lactate concentration.

Figure 4a shows the device positioned on the top of a cylindrical optical phantom. The dimensions of the phantom are sufficiently large that its light absorption and scattering can be assumed to occur within a homogeneous tissue without boundaries. To mimic sweat on skin, a small volume (50 μL) of lactate solution in PBS (pH 6.0) was dropped onto the phantom before placing the integrated device on top. Figure 4b shows the real-time readout of the device in response to different lactate concentrations (0 mM, 10 mM, 40 mM, and 80 mM). With the lactate concentration increases, the normalized absorption markedly rises more rapidly over time and saturates at a higher value. This behavior is anticipated, as elevated lactate concentrations induce faster generation and accumulation of TMB_ox_ in the hydrogel, leading to a rapid increase in its optical absorption at both 660 nm and 905 nm. We took use of both wavelengths to eliminate background noise and baseline fluctuations. While responding significantly to lactate, the device showed highly stable concurrent readings of tissue oxygenation levels (StO_2_) and BVI at all lactate concentrations (Figure 4c,d). This stability is crucial for guaranteeing that the integrated device can precisely and simultaneously monitor multiple parameters (sweat lactate, tissue oxygenation, and tissue blood volume) without cross-sensitivity between the results, making our device first of its kind and potentially addressing critical challenges in applications such as exercise physiology monitoring and sepsis management.

### 3.6. On-Body Measurements of Sweat Lactate

**Figure 5:**
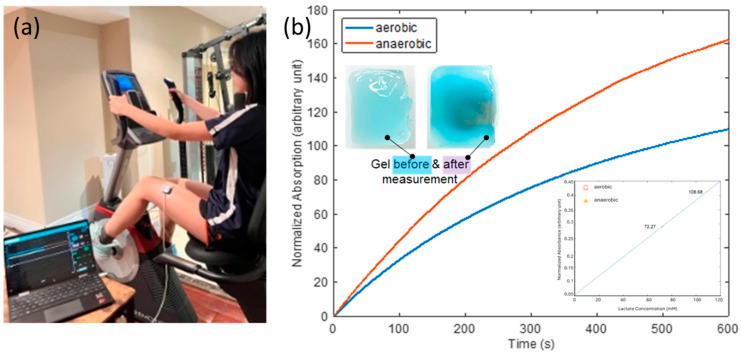
(**a**) Picture of the test setup showing the device worn on the researcher’s left thigh while on a stational bike, with the laptop running data collection software. (**b**) Normalized absorption vs. time response curves for the aerobic and anaerobic conditions. Inset-left up: appearance of gel before and after the test; inset-right down: lactate detection calibration curve.

The integrated device’s ability to monitor sweat lactate under aerobic and anaerobic conditions was evaluated. Figure 5a shows the experimental setup, in which the device was worn on the left thigh of a researcher using a medical-grade adhesive, while exercising on a stationary bike. The optical electronics are part of medical devices that have received regulatory approval for human wear and the alginate-based hydrogel is biocompatible, ensuring no harm to human skin or tissue. The device was connected to a laptop via a flexible cable, plugged into its USB port.

The researcher followed a two-phase exercise protocol that included both aerobic and anaerobic exercises. The aerobic phase involved light-intensity exercise to induce moderate perspiration. This was followed by the anaerobic exercise phase, which consisted of a 10-min graded incremental exercise test (GXT) on a stationary bike [50]. In the GXT protocol, the starting exercise intensity level (*W_start_*) was set at one-fourth of the maximum exercise intensity (*W_max_*), expressed as *W_start_* = *W_max_*/4. The exercise intensity increased incrementally each minute, with the incremental change (Δ*W*) calculated as Δ*W* = (*W_max_* − *W_start_*)/8. The exercise intensity at any given time *t* during the test was defined by the equation *W*(*t*) = *W_start_* + Δ*W*·*t*. The researcher continued this incremental exercise until reaching the maximum intensity level (*W_max_*).

The recorded normalized optical absorption curves are shown in Figure 5b. The starting times (t = 0) on the curves were immediately after the completion of the aerobic and anaerobic phases, respectively. The device’s response was markedly more pronounced under anaerobic conditions, as evidenced by the steeper increase in absorption. This was expected, as lactate generation is known to elevate in anaerobic conditions, presumably due to heightened glycolysis in the absence of oxygen. This result indicates that our device can differentiate the aerobic condition, with ample oxygen availability, and anaerobic conditions, characterized by restricted oxygen levels, by monitoring sweat lactate. The inset in the upper left of Figure 5b shows images of the lactate-sensing hydrogel before the device was placed on the researcher and after it was removed following the completion of the whole two-phase exercise protocol. Prior to lactate exposure, the hydrogel exhibited a lighter hue, however, following the test, it becomes more saturated, signifying its interaction with lactate over time. This visual alteration aligns with the absorption data, substantiating the notion that lactate concentration directly influences the optical absorption of the gel.

Furthermore, the quantitative determination of lactate concentration was attempted. A calibration curve was generated using the experimental data gathered on the optical phantom. The initial slopes (first 100 s) of the response curves in Figure 4b were found to be linearly proportional to lactate concentration, as shown in the lower right inset of Figure 5b. Calibration curves were established using a standardized volume of 50 µL, as this ensures uniform and complete coverage of the sensing gel surface. Note that the gels were fully hydrated, therefore, no swelling is expected upon contact with sweat. Equilibrium-driven lactate diffusion into the sensing gel is anticipated, rather than a net inflow of sweat. In practical situations, the initial rate of response was used to determine lactate concentration, regardless of sweat volume, assuming that sufficient sweat uniformly covers the surface of the sensing hydrogel, as is the case during the generation of calibration curves. The design of the system inherently addresses normalization against perspiration volume, as the colorimetric signal produced depends on lactate concentration rather than the absolute volume of sweat absorbed. The hydrogel matrix’s capacity and geometry were specifically designed to maintain consistent enzymatic reaction dynamics and diffusion over time, thus mitigating the effects of fluctuating perspiration volumes within a practical range.

By extracting the slopes of the aerobic and anaerobic curves in the first 100 s, sweat lactate levels were determined to be 72 mM and 109 mM after aerobic and anaerobic exercises, respectively, validating the correlation between elevated sweat lactate level and cardio hypoxia. This experimental setup and our results demonstrated the device’s practical applicability in physical activity, emulating real-world scenarios like fitness monitoring or clinical diagnostics.

### 3.7. On-Body Continuous Monitoring of Multiple Parameters

**Figure 6:**
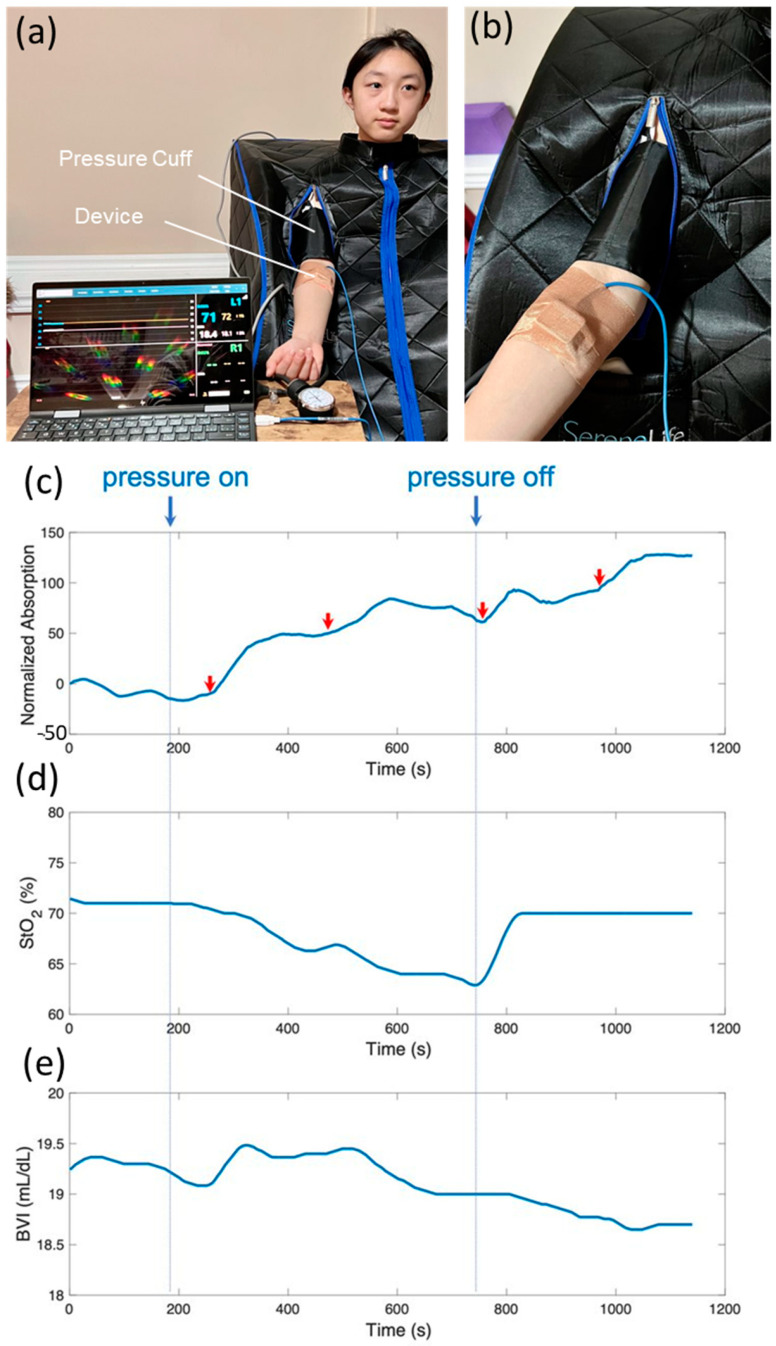
(**a**) Picture of a student researcher sitting in a sauna tent to stimulate sweating while a pressure cuff was used to create temporary hypoxia in the forearm. (**b**) A zoomed-in view of the pressure cuff worn on the upper arm and the wearable device on the forearm. (**c**–**e**) Simultaneous monitoring of sweat lactate production (top panel), tissue oxygenation level (middle panel), and BVI (bottom panel).

Figure 6a depicts an experimental setup where a researcher was seated within a sauna tent to stimulate perspiration. A pressure cuff affixed to the upper right arm was used to induce localized and temporary hypoxia in the forearm (Figure 6b) in a controlled manner, while the device concurrently measured local sweat lactate, tissue oxygenation and tissue blood volume (Figure 6c–e). The pressure-cuff protocol is a clinically safe and well-established method for creating blood flow restriction [51].

Figure 6c shows the normalized absorption by the lactate-sensing hydrogel over time. A distinct rise in lactate was observed after the onset of pressure at around 200 s, along with multiple lactate spikes during the pressure-on period, indicated by red arrows in Figure 6c, where steep rises in optical absorption were recorded. Concurrently, the device captured the drastic decrease and recovery of StO_2_ in response to the pressure being applied and released, respectively (Figure 6d). Figure 6e shows that tissue blood volume increased initially in response to the onset of pressure, and then gradually decreased to normal. Our results are in excellent agreement with the physiological changes expected during blood flow restriction exercise using a pressure cuff. The initial increase in tissue blood volume is due to the sudden full restriction of venous outflow and partial restriction of arterial inflow. The blood inflow and outflow were later balanced. The applied pressure created local hypoxia in the skin and tissue underneath the device, as confirmed by the measured StO_2_ levels. Sweat lactate is produced by local metabolism in sweat glands and surrounding tissues. Consequently, sweat lactate spikes were observed coinciding with the drop in StO_2_ levels, indicating local hypoxia. While tissue oxygenation recovered immediately after the release of pressure, elevated sweat lactate production persisted for about 5 min after the release of pressure, at which point the optical absorption of the hydrogel plateaued. It indicates that sweat lactate is a good indicator of cellular hypoxia, which is related to tissue oxygenation but distinctly different physiologically.

Table 1 presents a comparative examination of the analytical performance of modern lactate detection systems across different sensor types, detection methodologies, and specimen kinds. Electrochemical sensors, specifically amperometric and voltametric techniques, prevail in the domain, with detection ranges extending from 0 to 1000 mM in blood serum and more focused ranges of 0 to 220 mM in sweat. These sensors are recognized for their swift response times and comparatively low expense. Nonetheless, they are often constrained by specificity, as certain sensors, particularly those employed for brain and peripheral tissue [52], demonstrate restricted linear ranges (0 to 3 mM), potentially affecting their adaptability in various physiological contexts [53]. Colorimetric methods, including those utilizing organic electrochemical transistors (OECT) and colorimetric combined with NIR techniques [54], demonstrate potential with reduced detection ranges, particularly in sweat (0–1 mM for OECT and 0–80 mM for the current study), underscoring their applicability for wearable non-invasive monitoring. Chemical detection methods, such as HILIC-ELSD and UV-Vis [55], provide commendable sensitivity and specificity; however, they are constrained by intricate sample preparation and elevated detection limits, generally in the microgram per milliliter (µg/mL) range, rendering them less appropriate for continuous real-time monitoring [56]. The present study’s integrated colorimetric and NIR detection technique provides a harmonious method, merging the sensitivity of NIR with the simplicity of colorimetric alterations, facilitating efficient detection in sweat and presenting a feasible solution for non-invasive lactate monitoring. The comparative research reveals the trade-offs among sensitivity, detection range, and application in various physiological fluids, highlighting the possibility for future developments in wearable, non-invasive lactate detection.

## 4. Conclusions

This study presented a comprehensive wearable system that can concurrently detect sweat lactate, tissue oxygenation, and tissue blood volume in a non-invasive and continuous fashion. This apparatus amalgamates two contemporary sensor technologies: a hydrogel-based colorimetric assay and a near-infrared sensor, within a compact design. The optical electronics within the gadget fulfill two functions: assessing tissue oxygenation and delivering a quantitative output of the colorimetric assay. The hydrogel-based colorimetric assay was initially assessed using UV-vis spectroscopy to adjust its composition for enzyme (LOX and HRP), chromogenic dye (TMB), and polymer (alginate) concentrations. The hydrogel exhibited sensitivity, biocompatibility, and stability. The variation in optical absorbance at 660 nm and 905 nm was directly related to sweat lactate content. The complete gadget was later evaluated on an optical tissue phantom and on a researcher’s leg while exercise on a stationary bicycle. The device accurately detected sweat lactate while consistently monitoring tissue oxygenation and distinguishing between aerobic and anaerobic states. The system was ultimately verified for on-body continuous monitoring of sweat lactate, a biomarker of cellular hypoxia, alongside tissue oxygenation and tissue blood volume, which signify tissue blood and oxygen supply, by employing a pressure cuff for blood flow restriction.

Although increased sweat lactate has been recognized as a potential marker for the early identification of hypoxia, facilitating timely intervention to avert substantial tissue damage, it lacks specificity. Besides hypoxia, many variables, including heat stress and dehydration, can also lead to increased sweat lactate levels. The simultaneous presence of increased sweat lactate and inadequate tissue oxygenation can distinctly indicate the initiation of anaerobic metabolism induced by hypoxia. Currently, no technology is available that can concurrently monitor both sweat lactate and tissue oxygenation levels, as integrating these two measurements without interference in a compact, wearable format has proven to be exceedingly difficult. The device examined in this study effectively resolved this difficulty. It possesses numerous potential applications in therapeutic settings, particularly for monitoring patients with conditions marked by compromised oxygen transport or metabolic irregularities. Furthermore, the technology could benefit athletes, astronauts, or individuals exposed to high altitudes, where rapid detection of hypoxia is crucial to prevent adverse health effects.

## Figures and Tables

**Table 1:** Comparative analysis of the analytical performance of contemporary lactate detection devices.

Sensor Type	Detection Method	Linear Range (mM)	Specimen	Ref.
Electrochemical	Amperometric	0–1000	Blood serum	[53]
Electrochemical	Amperometric	0–3	Brain and peripheral tissue	[57]
Electrochemical	Voltametric	10–250	Serum	[58]
Electrochemical	Amperometric	1–220	Sweat	[52]
Electrochemical	mperometric	1–50		[59]
OECT	Colorimetric	0–1	Sweat	[54]
Electrochemical	Amperometric	0–24	Sweat	[60]
Chemical	HILIC-ELSD	20–400 µg/mL	Human milk	[55]
Chemical	UV-Vis	0.1–1.7	PBS	[56]
Chemical	Colorimetric + NIR	0–80	Sweat	This work

## Data Availability

Data will be available on reasonable request to the corresponding author.
